# Histological properties of oscillating intracardiac masses associated with cardiac implantable electric devices

**DOI:** 10.1002/joa3.12346

**Published:** 2020-04-20

**Authors:** Yasuo Miyagi, Yasuhiro Kawase, Shinobu Kunugi, Hiroya Oomori, Takashi Sasaki, Shun‐ichiro Sakamoto, Yosuke Ishii, Tetsuro Morota, Takashi Nitta, Akira Shimizu

**Affiliations:** ^1^ Department of Cardiovascular Surgery Nippon Medical School Tokyo Japan; ^2^ Department of Analytic Human Pathology Nippon Medical School Tokyo Japan

**Keywords:** CIED, histology, IE, intracardiac mass, vegetation

## Abstract

**Background:**

There have been a few cases of echogenic cardiac implantable electric device (CIED) lead‐associated oscillating intracardiac masses (ICMs) in leads imaged by echocardiography. The histological properties of ICMs could help clarify the etiological diagnosis. Although there is extensive literature on mass size, the histological properties of such masses have not been characterized. The aim of this research was to clarify the histological features of oscillating ICMs in CIED patients.

**Methods:**

Preoperative echocardiography was performed in all candidates for CIED removal. In the patients with ICMs, specimens were obtained by 3 methods: direct tissue collection during open‐heart surgery; tissue collection together with the CIED lead during transvenous extraction; and tissue collection by catheter vacuum during transvenous CIED removal. A standard histopathological examination of ICM tissue was performed.

**Results:**

A total of 106 patients underwent lead removal in our institute (April 2009‐March 2018); 14 patients had an ICM (13.2%), and 7 specimens were obtained in patients with CIED lead‐related ICM. Following histological examination, 2 types of ICM were identified: one mainly composed of thickened endocardium (EN type; 3 patients), and the other mainly an aggregate of inflammatory cells as a neutrophil cell (NC type; 4 patients).

**Conclusions:**

Two histological types of intracardiac masses, including a thickened endocardium type and a neutrophil cell type, were identified. These classifications might help make an accurate histological diagnosis of lead‐associated intracardiac masses.

## Graphical abstract

This research was to clarify the histological features of oscillating ICMs in CIED patients. Two histological types of intracardiac masses, including a thickened endocardium type and a neutrophil cell type, were identified. These classifications might help make an accurate histological diagnosis of lead‐associated intracardiac masses.

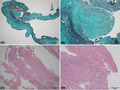

## 1 INTRODUCTION

On the basis of clinical trials including large populations of patients with cardiac implantable electrical devices (CIEDs), the number of CIED patients has significantly increased over the last decade.^1^ However, candidates requiring removal of CIED systems have also been increasing at an astounding rate.^2^, ^3^, ^4^ The reasons for removal in the majority of these candidates were lead dysfunction, cardiac device‐related endocarditis (CDRIE), and local device infection including pocket infection.^5^, ^6^ In particular, CDRIE has the potential to increase mortality and morbidity; therefore, prompt recognition and management of CDRIE may improve patient outcomes. According to the 2017 HRS statements on lead management, the presence of an echocardiographic right‐side intracardiac mass (ICM) on CIED leads is critical for the accurate diagnosis of CDRIE, determining the need for CIED system removal.^2^

With the development of echocardiography (ie, transesophageal echocardiography [TEE]), the incidence of CIED lead‐associated masses has been reported to be 1%‐14% as endocarditis and as incidental detection on echocardiographic studies (transthoracic echocardiography [TTE] and TEE).^5^, ^7^, ^8^, ^9^

In the clinical setting, the determination of the etiology of an echogenic mass remains challenging. There are not a few patients with echogenic ICMs without systemic infection or venous thromboembolism.^10^ Today, the main clinical question is whether an ICM represents a thrombus or vegetation, but distinguishing a vegetation from a thrombus is difficult in clinical practice. Therefore, the histological characteristics of ICMs need to be evaluated. However, the histological properties of intracardiac masses have not been investigated. Understanding the histological characteristics of ICMs may help clarify their etiology.

The purpose of this investigation was to further elucidate the histological origins of ICMs in CIED patients.

## 2 METHODS

### 2.1 Patient population and data collection

This retrospective investigation included all patients who had undergone lead and device removal in the Department of Cardiovascular Surgery at Nippon Medical School Hospital between April 1, 2009 and March 31, 2018. All procedures were performed according to the guidelines of the Declaration of Helsinki^11^ and were approved by the Ethics Committee of the Nippon Medical School in Tokyo, Japan (reference number 30‐11‐1043). All data concerning preoperative clinical presentation, microbiological findings, echocardiographic data, and histopathological diagnosis were collected from these records. The indications for device removal and the procedure for lead and device removal followed the 2009 and 2017 Heart Rhythm Society consensus.^3^, ^4^ Pathologists performed histopathological examinations of the specimens after ICM removal.

### 2.2 Histological evaluation of intracardiac masses and specimen collection

Specimens were obtained by 3 methods: (a) direct ICM tissue collection during open‐heart surgery for CIED removal; (b) ICM tissue collection together with CIED lead during transvenous extraction; and (c) ICM tissue collection by catheter vacuum during transvenous CIED removal. A standard histopathological examination of ICM tissue was performed, and the extracted tissues were immediately fixed in 10% formalin. Tissues were then embedded in paraffin wax and later stained with hematoxylin‐eosin and/or Masson trichrome. Pathologists performed histopathological examinations of all specimens.

### 2.3 CIED system removal procedure

The decision to perform transvenous removal or open‐heart surgery was made during a treatment team meeting. Required criteria for open‐heart surgery included an ICM length greater than around 30 mm, associated left‐side endocarditis, and included perforated leads. Both transvenous procedures and open‐heart surgery were performed in the operating room with the patient under general anesthesia. All patients were monitored via transesophageal and intracardiac echocardiography, in addition to general cardiac monitoring. Percutaneous cardiopulmonary support was also on standby in the operating room throughout the procedure.

#### 2.3.1 Transvenous procedure

Standard methods for transvenous lead extraction were performed. The leads were extracted using the subclavian approach. Laser‐assisted lead extraction was attempted through an excimer laser sheath (SLS II or GL, DVX) with a locking stylet.

Following transvenous lead extraction, some of the ICMs were remained in the right atrium. The remaining ICM was suctioned using an aspiration catheter (Launcher 8‐Fr, Medtronic) with the area accessed via a femoral vein approach. Several specimens were obtained using this method.

#### 2.3.2 Open‐heart surgery

During open‐heart surgery, extracorporeal circulation was introduced through a median sternotomy. Conventional bicaval cannulation was used during the open‐heart procedure. Both great veins had a tourniquet applied to control bleeding during the time of incision of the right atrium. The tips of the atrial and ventricular leads were removed directly through the right atrial incision. During or before cardiopulmonary bypass, an excimer laser sheath was used to ablate lead adhesions through the subclavian vein and the superior vena cava. A sheath was inserted from an antegrade subclavian vein or retrograde superior vena cava according to lead condition. ICMs and leads were removed entirely via the open‐heart procedure with laser sheath‐assisted lead extraction.

### 2.4 Echocardiographic study

All patients underwent TTE before surgery. A subset of patients also underwent TEE. An ICM was defined as an oscillating mass found on a lead and confirmed in multiple views by TTE or TEE. The location of the ICM was determined by echocardiography. ICM morphology (diameter and number) was also examined using the data obtained from the echocardiogram. The diameter was measured in the longest length of the ICM. The number of ICMs was counted in multiple echocardiogram views. Cardiologists or echocardiologists overviewed all echocardiogram data.

### 2.5 Statistical analysis

All continuous variables are expressed as means ± SD of the mean. All categorical variables are reported as numbers (%) of patients. A paired Student's *t* test was used to compare all the continuous variables. Categorical variables were analyzed using a chi‐squared test. Significance was accepted at *P* < .05. All statistical analyses were performed using SPSS version 22.0 (SPSS Inc).

## 3 RESULTS

### 3.1 Patient population

From April 2009 through March 2018, 106 patients underwent CIED removal with a total of 204 leads in our institute. In all, 14 patients had ICMs with echocardiographic evidence in this series (13.2%). Of these patients, 11 (10.3%) had CIED lead‐related ICMs. The other 3 CIED patients (3%) had solitary left‐side cardiac vegetations (ie, aortic or mitral valve).

### 3.2 Histological evaluation of intracardiac masses

**FIGURE 1:**
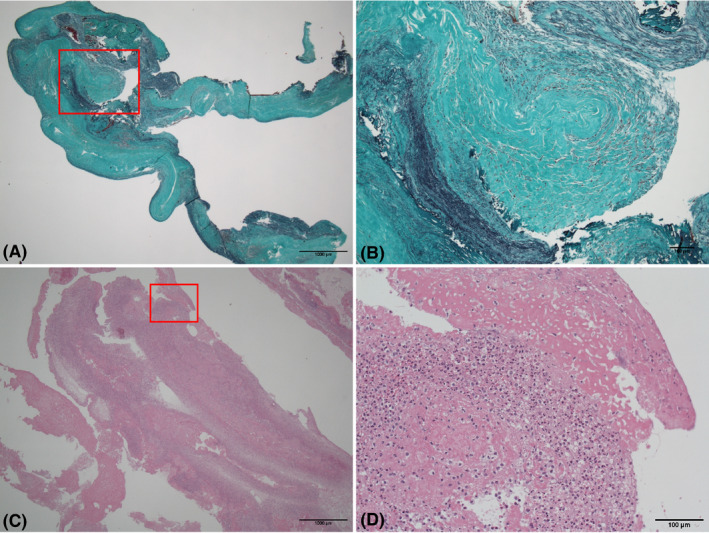
Histological findings of intracardiac masses and classification. A, The distinctive endocardium (EN) type is shown. Small polypoid intracardiac mass (ICM) lesion with thickened elastofibrotic endocardial tissue. Elastic‐Masson‐Goldner (EMG) stain. Bar = 100 μm. B, High‐power field of EN type ICM. EN type ICM is mainly composed of collagen fibers. EMG stain. Bar = 100 μm. C, The distinctive neutrophil cell (NC) type is shown. The NE type ICM is formed from inflammatory cells, mainly neutrophil cells. Hematoxylin‐eosin stain. Bar = 100 μm. D, High‐power field of NE type ICM. Fresh and organized fibrin deposition with degenerated inflammatory cells (neutrophil cells) infiltrated into an NC type ICM. Hematoxylin‐eosin stain. Bar = 100 μm

Seven specimens were obtained from patients with CIED lead‐related ICMs. Analysis of the histological features of the ICMs showed that there were 2 major histological types (Figure 1). The first type was composed of fibrous tissue whose principal component was thickened endocardium (endocardium type; EN type). Figure 1A,B shows the distinctive findings of EN type with Elastic‐Masson‐Goldner (EMG) stain. The mass had polypoid collagenous tissue protruding from the endocardium. The second type was primarily composed of an aggregate of neutrophils as inflammatory cells and/or fresh fibrin (neutrophil cell type; NC type). Figure 1C,D shows the typical structure of the NC type formed mainly from fresh fibrin with inflammatory cell infiltration, consisting primarily of neutrophil cells, with hematoxylin‐eosin stain. Three patients had EN type ICMs, whereas the other 4 patients had NC type ICMs on histological analysis. Interestingly, all ICM samples had negative results with Giemsa and Gram stains for bacterial culture. Three specimens were directly collected during open‐heart surgery (EN type = 1, NC type = 2), and the other 4 specimens were collected during transvenous lead extraction. Two ICMs were obtained together with extracted leads because they were rigidly attached to the leads (EN type = 2). Tissues from the other 2 ICMs were sampled using catheter vacuum after removal of the transvenous lead (NC type = 2).

### 3.3 Patients' demographics

The demographics of the patients with the 2 types of ICM are summarized in Table 1. Their demographics were similar with respect to age (*P* = .73), gender (*P* = .27), and type of CIED (*P* = .66). Concerning the type of CIED, pacemakers were implanted in 3 patients (EN type = 1 and NC type = 2). ICDs were implanted in 4 patients (EN type = 2 and NC type = 2). The details of the leads are shown in Table 1. The lead number was similar in both types (EN type vs NC type = 1.7 ± 0.6 vs 2.6 ± 0.8, *P* = .12), although the NC type had a tendency to be implanted in the patient longer (EN type vs NC type = 4.8 ± 0.8 years. vs 12.2 ± 7.5 years, *P* = .08).

**TABLE 1:** Patients’ demographics

	EN type, N = 3	NC type, N = 4	*P* value
Age, y	62.7 ± 19.7	68.3 ± 5.0	.73
Gender, male, %	33.3	75.0	.27
Primary disease	Brugada syndrome: 1	SSS: 1	—
	SSS: 1	CAVB: 1	
	HCM: 1	DCM: 1	
		VT: 1	
PM/ICD	½	2/2	.66
Total lead number	1.7 ± 0.5	2.6 ± 0.8	.12
RA lead	2	4	
PM/RV lead	1	4	
ICD/RV lead	2	2	
LV lead	0	1	
Lead age, y	4.8 ± 0.8	12.2 ± 7.5	0.08

Note

Values are means ± SD or N (%).

Abbreviations: CAVB, complete atrioventricular block; DCM, dilated cardiomyopathy; HCM, hypertrophic cardiomyopathy; ICD, implantable cardioverter defibrillator; LV, left ventricle; PM, pacemaker; RA, right atrium; RV right ventricle; SSS, sick sinus syndrome; VT, ventricular tachycardia.

The clinical presentations are summarized in Table 2. Concerning the indications for CIED removal, all cases had class I or IIa indications for lead extraction due to ICMs with or without signs of infection.^3^, ^4^ There was 1 infected (fever unknown origin (FUO): 1) patient and 1 non‐infected patient (lead failure: 1) in the EN type. Otherwise, all patients showed indications of infection (pocket infection: 2, FUO: 2) in the NC type. Local and systemic signs of infection were not different between the 2 types. Concerning positive blood cultures, all NC type patients had positive results (*Staphylococcus aureus*: 1, *Staphylococcus epidermidis*: 3). On the other hand, no EN type patients had a positive blood culture.

**TABLE 2:** Clinical presentation

	EN type, N = 3	NC type, N = 4	*P* value
Indication for lead extraction	Lead failure: 1 Increasing mass size: 1 FUO: 1	Pocket infection: 2 FUO: 2	—
Local infection signs	0	2	.22
Systemic infection signs	1	2	.66
Positive blood culture	0	4	.008

Note

Values are means ± SD or N (%).

Abbreviations: FUO, fever of unknown origin.

### 3.4 Echocardiographic study

On echocardiographic study, the ICMs related to the right‐sided leads were evaluated, and their morphological characteristics (shape, number, size, and location) and qualitative features (echogenicity) were assessed.

#### 3.4.1 ICM morphology: shape, number, size, and location

**FIGURE 2:**
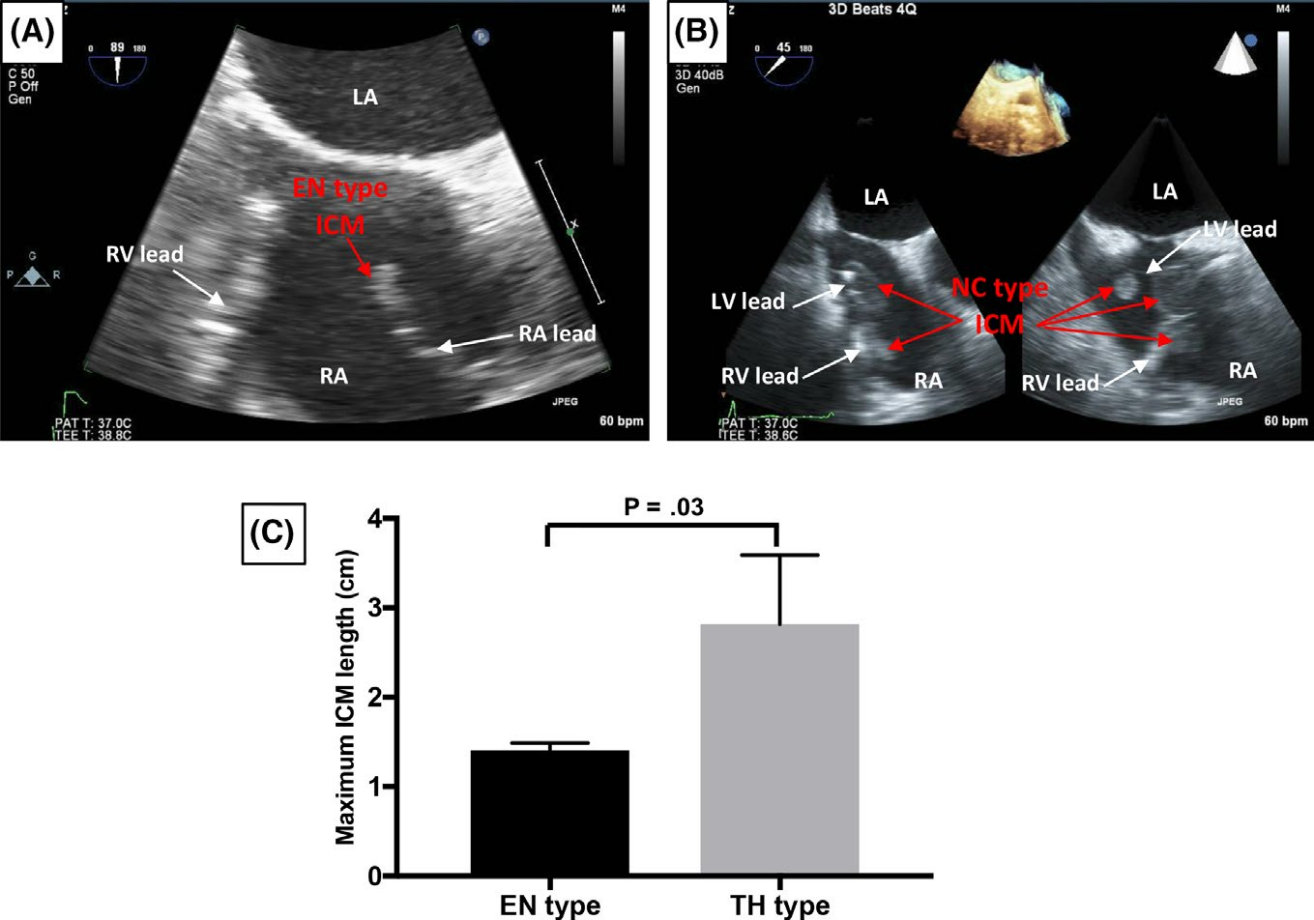
Echocardiographic study of EN type intracardiac mass (ICM) and NC type ICM. A, The EN type ICM is shown. The typical EN type ICM appears as a solitary caulescent round shape. B, The NC type ICM is shown. The typical NC type ICM appears as a multilocular lobular shape. There are multiple ICMs in this view. C, Comparison of ICM maximum length between EN type and NC type ICMs. The NC type is significantly longer (2.8 ± 0.7 cm) than the EN type (1.4 ± 0.1 cm, *P* = .03). Abbreviations: RA, right atrium; LA, left atrium; RV, right ventricle; LV, left ventricle

Intracardiac mass shapes were classified as solitary round in shape, multilocular lobular types, and mixed types (Figure 2A,B). All EN type ICMs were observed as solitary caulescent round shapes (Figure 2A). On the other hand, in the NC type, all ICMs had multilocular lobular shapes (Figure 2B). Two patients had a solitary mass, whereas the other patients showed signs of multiple ICMs. Some of the NC type patients had ICMs with strips.

**FIGURE 3:**
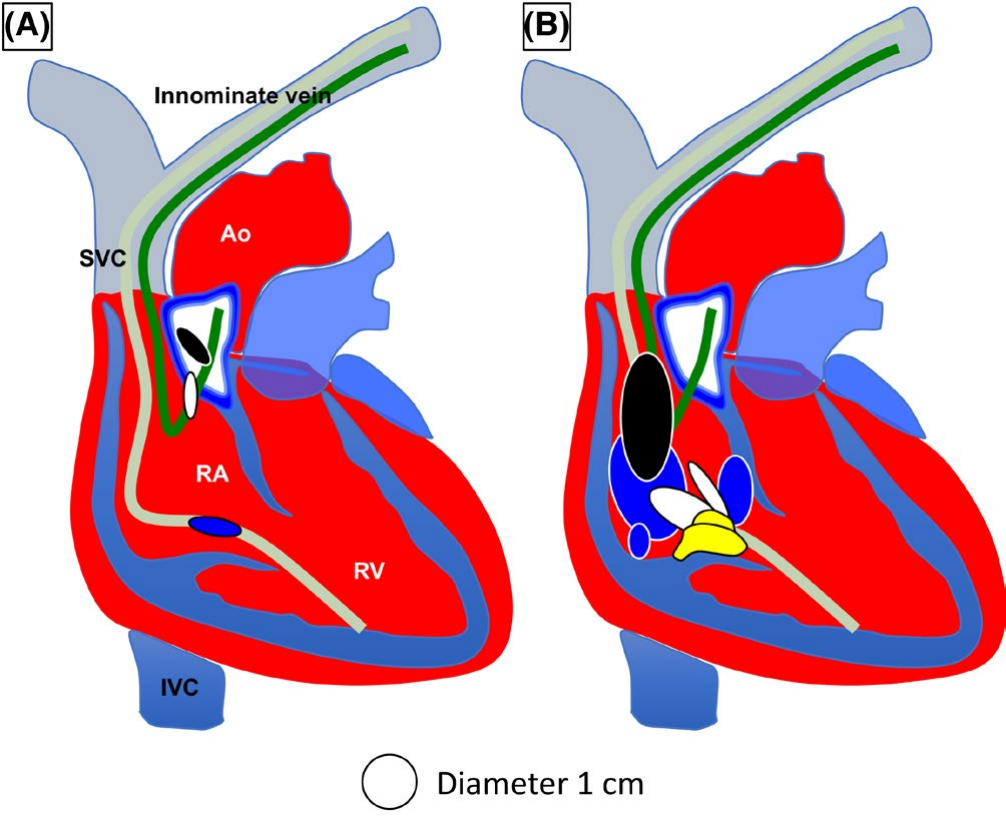
Location of intracardiac masses (ICMs) in the heart. A, EN type ICM locations are shown. The different color (white, black, and blue) circles correspond to 3 individual cases. All EN type ICMs are located in the right atrium with CIED leads and the tricuspid valve orifice. B, NC type locations are shown. The different color (white, black, blue, and yellow) circles correspond to 4 individual cases. The majority of NC type ICMs are located in the right atrium. One NC type ICM is located in the right ventricle below the tricuspid valve orifice. Abbreviations: RA, right atrium; LA, left atrium; RV, right ventricle; LV, left ventricle; Ao, aorta; SVC, superior vena cava; IVC, inferior vena cava. The dark green line indicates the atrial lead. The light green line indicates the ventricular lead.

Comparing the maximum lengths of the ICMs, the NC type was significantly longer (2.8 ± 0.7 cm) than the EN type (1.4 ± 0.1 cm, *P* = .03, Figure 3C). Concerning ICM location, EN type ICMs were in the right atrium on multiple echocardiogram views. Two EN type ICMs were attached to atrial leads (Figure 3A, black and white mass). The other EN type ICM was attached to the ventricular lead and tricuspid orifice (Figure 3A, blue mass). In the NC type, most ICMs were in the right atrium (Figure 3B). An ICM was also identified in the right ventricle (Figure 3B, yellow mass). The other ICM was located at the superior vena cava and attached over the RA and RV leads (Figure 3B, black mass). In one of the patients who had multiple ICMs, the ICMs were located at the center of the right atrium, the superior vena cava, and the coronary sinus ostium (Figure 3B, blue masses).

## 4 DISCUSSION

This study showed 2 histological types of CIED lead‐related ICMs. The main structure of the first type was composed of layered elastic tissue, which was defined as thickened endocardium (EN type, Figure 1A,B). The second type was principally formed by inflammatory cell infiltration, primarily neutrophils, with fibrin on hematoxylin‐eosin staining (NC type, Figure 1C,D). Although the patients with the 2 types had similar clinical backgrounds, all NC type patients had positive blood cultures before the operation. Conversely, there were no patients with positive blood cultures in the EN type.

The primary structures of the EN type ICMs consisted of thickened endocardium. Considering these histological features, the EN type ICM might be related to a fibrous sheath covering the CIED leads. After the implantation of the leads, fibrin deposits around the leads. This fibrin ultimately forms into connective tissue resulting in full coverage of the leads.^10^, ^12^, ^13^, ^14^ It might be highly likely that the so‐called “ghosts” are the same histological strain as the EN type ICM.^15^ “Ghosts” are detected as residual floating fibrous tissue after transvenous lead extraction by echocardiography. In EN type ICMs, 2 cases were detected before lead extraction, and the other one was observed after lead extraction around the tricuspid valve orifice. Two of EN type ICMs were at in the cavity with right atrial leads and the other one was placed on tricuspid orifice (see Figure 3; left panel). We speculate the outgrowth process of the floating EN type ICM. In the right atrium, the prevalent atrial blood flow pattern is vortical flow and/or vortices flows.^16^ Vortical flow caused low wall shear stress.^17^ The leads and the fibrosis sheath were exposed by vortices and low wall shear stress. Low shear stress causes endothelial cells injury on lead surface fibrotic tissue and provokes the neointimal hyperplasia.^18^, ^19^ In line with this process, hyperplastic fibrosis grows on the endocardial surface and into the RA cavity as the EN type ICMs.

On the other hand, the main structure of NC type ICMs was inflammatory cells, mainly neutrophils, with fibrin formation. In addition, the specimens of the NC type ICMs were obtained from patients with bacteremia (*Staphylococcus aureus*: 1, *Staphylococcus epidermis*: 3). These histological features and clinical scenarios suggested the possibility that NC type ICMs were related to infective endocarditis. The substance of NC type ICMs could be “vegetation.” During the vegetation‐generating process, bacteria bind to coagulum and form colonies. Bacteria, inflammatory cells, platelets, and adhesion molecules (ie, fibronectin) have effects on the growth of vegetation. These factors were identified in the histological findings of NC type ICMs.^20^, ^21^, ^22^

In clinical practice, the major question is whether an ICM represents a thrombus or vegetation.^10^ In other words, the question is whether an ICM represents infection or not. In this regard, quite different therapeutic strategies are selected between a thrombus and a vegetation. In the case of “vegetation” with a CIED lead, invasive therapy with lead extraction and longer antibiotic treatment are required. Recently, a new biopsy technique for ICM‐attached CIED leads was reported in order to provide a more accurate differential diagnosis between a thrombus and vegetation.^10^ ICM tissues collected by biopsy were evaluated by histology. On the basis of histological findings, thrombus and vegetation could be clearly differentiated. The present histological data may be helpful for more precise diagnosis of ICMs related to CIED leads. Although a fresh thrombus type ICM was not seen in the present case series, oscillating EN type ICMs were detected on preoperative echocardiography. This study may also suggest that EN type ICM should be added to the differential diagnosis of ICM with CIED leads. The EN type ICMs were categorized into the non‐infection type, the same as thrombus.

Interestingly, all ICM samples had negative results with Giemsa and Gram stains for bacterial culture. There may be some reasons for negative results for bacterial culture in this series. As our institute is referral hospital, the patients were referred from previous hospital and all patients underwent antibiotics therapy before CIED device removal. Preceded antibiotics therapies might take effect on the culprit pathogens before the operation. As a result, the negative findings of Giemsa and Gram stains were observed in histological examinations.

The morphological features of EN type and NC type ICMs were examined using echocardiography. The shapes of the ICMs were different between EN and NC types. Considering the histological features, multilocular lobular shapes in the NC type ICMs might be generated by low blood flow through the right atrium and soft fibrin formation. Round shapes in the EN type ICM were also formed by exposure of the bloodstream to fibrotic tissue. The size of ICMs was significantly different between the EN type and the NC type. The difference in size may be based on their distinct growth durations. The EN type made by endocardial tissue, like encapsulated fibrosis, grows slowly, showing monthly changes.^12^, ^23^ However, the NC type made mainly by fibrin can develop day‐by‐day in bacteremia.^20^, ^24^

### 4.1 Study limitations

This investigation included a small subset of CIED patients. It is possible that the numbers of the patients in EN type and NE type might be too small for the statistical analysis. However, the limited number of patients was due to the small population of ICM patients. The incidence of ICM patients in all echocardiographic studies was reported to be 1%‐14%.^5^, ^7^, ^8^, ^9^ In our cohort, 14 patients (13.2%) had ICMs with echocardiographic evidence. The number is needed to increase for more effective statistical evaluation in the future. Future investigations should increase the number of patients included in the analysis to provide further insights into the histological properties and challenges associated with ICMs.

## 5 CONCLUSIONS

This investigation used histopathological analysis to identify 2 types of ICMs in patients with CIEDs: a thickened endocardium type and an inflammatory cell type. These 2 types of ICMs showed distinct characteristics with respect to morphology, location, and number on echocardiographic assessment.

## CONFLICT OF INTEREST

All authors declare that there is no conflict of interest related to this study.
